# Case report: Nephrotic syndrome and portal hypertensive ascites after allogeneic hematopoietic stem cell transplantation: a rare manifestation of chronic graft-versus-host disease

**DOI:** 10.3389/fimmu.2024.1464616

**Published:** 2024-10-16

**Authors:** SanXi Ai, YuBing Wen, XiaoHong Fan, TianRui Hua, Wei Ye, XueMei Li, Yan Qin

**Affiliations:** ^1^ Department of Nephrology, Peking Union Medical College Hospital, Chinese Academy of Medical Sciences & Peking Union Medical College, Beijing, China; ^2^ Department of Medicine, Peking Union Medical College Hospital, Chinese Academy of Medical Sciences & Peking Union Medical College, Beijing, China

**Keywords:** chronic GVHD, membranous nephropathy (MN), thrombotic microangiopathy (TMA), ruxolitinib, ascites

## Abstract

Chronic graft-versus-host disease (GVHD) is a major complication after allogeneic hematopoietic stem cell transplantation (HSCT). Chronic GVHD may have atypical manifestations affecting non-classical organs. The diagnosis in patients with atypical manifestations of chronic GVHD is particullarly challenging, and there is a lack of knowledge regarding their pathogenesis and treatment. We reported a case who developed post-HSCT nephrotic syndrome and portal hypertensive ascites, which are both rare and atypical manifestations of chronic GVHD. Kidney biopsy revealed membranous nephropathy and renal thrombotic microangiopathy with glomerular immune deposits, suggesting antibody-mediated kidney injury. Treatment with ruxolitinib resulted in remission of both nephrotic syndrome and ascites, suggesting a role of cytokines in the pathogenesis. This case highlighted the awareness of nephrotic syndrome and portal hypertensive ascites as atypical manifestations of chronic GVHD, and the efficacy of ruxolitinib for the two manifestations.

## Introduction

Allogeneic HSCT is an established treatment for many hematological disorders. Chronic GVHD remains a serious and common complication of allogeneic HSCT, with a incidence of 30%-70% (1). Chronic GVHD may involve multiple organs, and has highly variable manifestations. Current NIH diagnosis criteria of chronic GVHD requires diagnostic or distinctive features in eight classical organs (1). However, it has been recognized that chronic GVHD may have atypical manifestations impacting non-classical organs (e.g. kidneys, endothelium, serositis) or atypical manifestations in classical organ systems (2). When an atypical chronic GVHD manifestation occurs in isolation without other diagnostic or distinctive chronic GVHD features, attribution to chronic GVHD is often a diagnosis of exclusion, which is particularly challenging because many other causes may complicate the differential diagnosis (2). These atypical manifestations of chronic GVHD significantly affect patient morbidity and mortality, however, there is a lack of knowledge regarding their pathogenesis, treatment and outcomes (2). Nephrotic syndrome and ascites are among the rare and atypical manifestations of chroinc GVHD (1, 2).

Ruxolitinib is a selective Janus kinases 1 and 2 (JAK1/JAK2) inhibitor, which can inhibit cytokines involved in GVHD. Efficacy of ruxolitinib against steroid-refractory chronic GVHD has been demonstrated in clinical trials. However, there are no previous reports on the use of ruxolitinib for the treatment of nephrotic syndrome or ascites after allogeneic HSCT. Herein, we report a patient who developed nephrotic syndrome and portal hypertensive ascites after allogeneic HSCT, with no NIH-defined chronic GVHD features. He was diagnosed with chronic GVHD and successfully treated with ruxolitinib.

## Case description

A 45-year-old male with HSCT was referred to our department for refractory ascites and nephrotic syndrome on August 2022. On December 2019, the patient developed fever, pancytopenia, hepatic dysfunction, and hepatosplenomegaly. He was diagnosed with Epstein-Barr virus (EBV)-associated hemophagocytic lymphohistiocytosis (EBV infection in natural killer cells), and received human leukocytes antigen full-match allogeneic HSCT from his brother on July 2020 with a conditioning regimen of cyclophosphamide, etoposide, and total body irradiation of 4.5 Gy. After transplant, he achieved remission of hemophagocytic lymphohistiocytosis. For prophylaxis of GVHD, he received cyclosporine, mycophenolate mofetil, and methotrexate until day +19 after transplant. On day +31 he developed acute skin and gastrointestinal tract GVHD, which was successfully treated with methylprednisolone (day +31 to day +46), cyclosporine (day +31 to day +90), and ruxolitinib (day +31 to day +104). During the first three months after transplantation, he had persistent normocytic anemia, slightly elevated ALP and GGT levels, with normal blood pressure, serum creatinine and urinalysis. Splenomegaly was persistently observed, without ascites or widening of the portal vein.

Four months after transplant when immunosuppressants had been discontinued, he developed abdominal distension and new-onset hypertension, with a maximum blood pressure of 165/119 mmHg. Ultrasound and CT scan showed splenomegaly, and new-onset gross ascites with widened portal vein. The ascitic fluid was sterile with a serum ascites albumin gradient of 23 g/L, suggesting portal hypertensive ascites. Urinalysis revealed proteinuria 1+ without hematuria, and 24-hour urinary protein was 0.17g, which increased to 1.65g three months later. Due to suspicion of tuberculous ascites, he was given a 4-drugs diagnostic anti-tuberculosis treatment for 14 months without any improvement, and he received recurrent drainage of ascites and intermittent diuretic therapy. His hypertension was controlled well by valsartan, but his proteinuria gradually progressed to nephrotic syndrome with serum creatinine increasing from 0.7 to 1.8 mg/dL.

Two years after transplant, he was referred to our department. Upon admission, his blood pressure was 135/85 mmHg. Complete blood count showed normocytic anemia without schistocytes in blood smears. Bone marrow examination revealed a hypocellular medulla. Liver function tests revealed elevated ALP, GGT, and AST, with normal bilirubin levels. Contrast-enhanced CT scan confirmed portal hypertension, without typical features of cirrhosis or hepatic venous occlusive disease (i.e. heterogeneous hypo-attenuation of liver parenchyma and patchy enhancement). Elevated tumor necrosis factor (TNF)-alpha and interleukin 6, and multiple serum antibodies were detected (**Supplementary Table S1**), but no connective tissue disease could be diagnosed. Serum anti-phospholipase A2 receptor (PLA2R) antibody was not detected. Screening for monoclonal protein, malignancy, and infection (treponema pallidum, hepatitis virus, human immunodeficiency virus, BK virus, paravirus B19, cytomegalovirus, EBV, adenovirus) were all negative. Results of laboratory findings at admission are listed in **Supplementary Table S1**.

**Figure 1:**
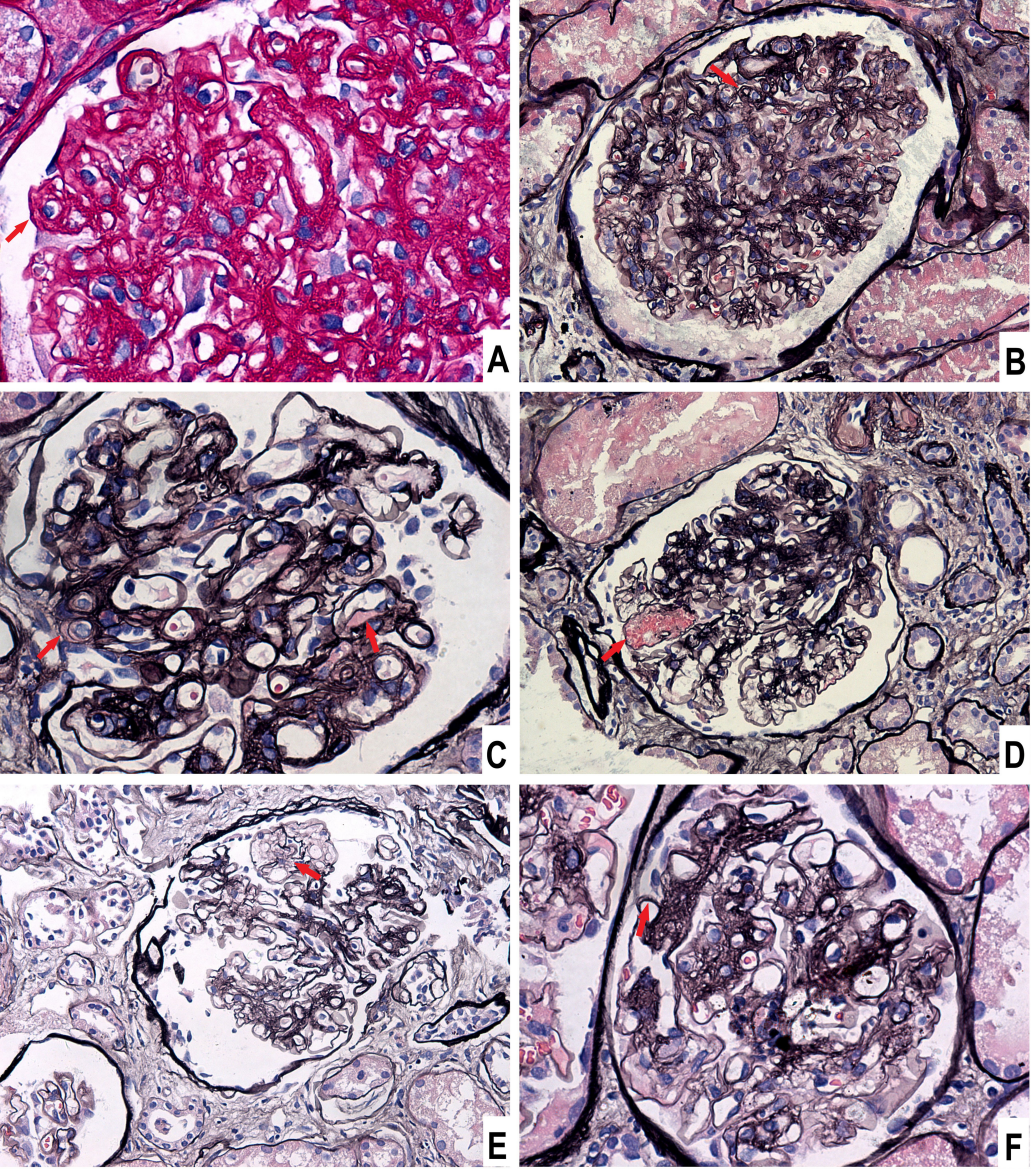
Light microscopic findings of glomeruli. **(A)** Endothelial swelling and subendothelial widening, Periodic acid-schiff stain, × 400. **(B)** Double contour of the glomerular basement membrane, Periodic acid-silver methenamine (PASM) stain, × 200. **(C)** Subendothelial exudation, PASM stain, × 400. **(D)** Microaneurysm, PASM stain, × 200. **(E)** Mesangiolysis, PASM stain, × 200. **(F)** Segmental spikes, PASM stain, × 400.

**Figure 2:**
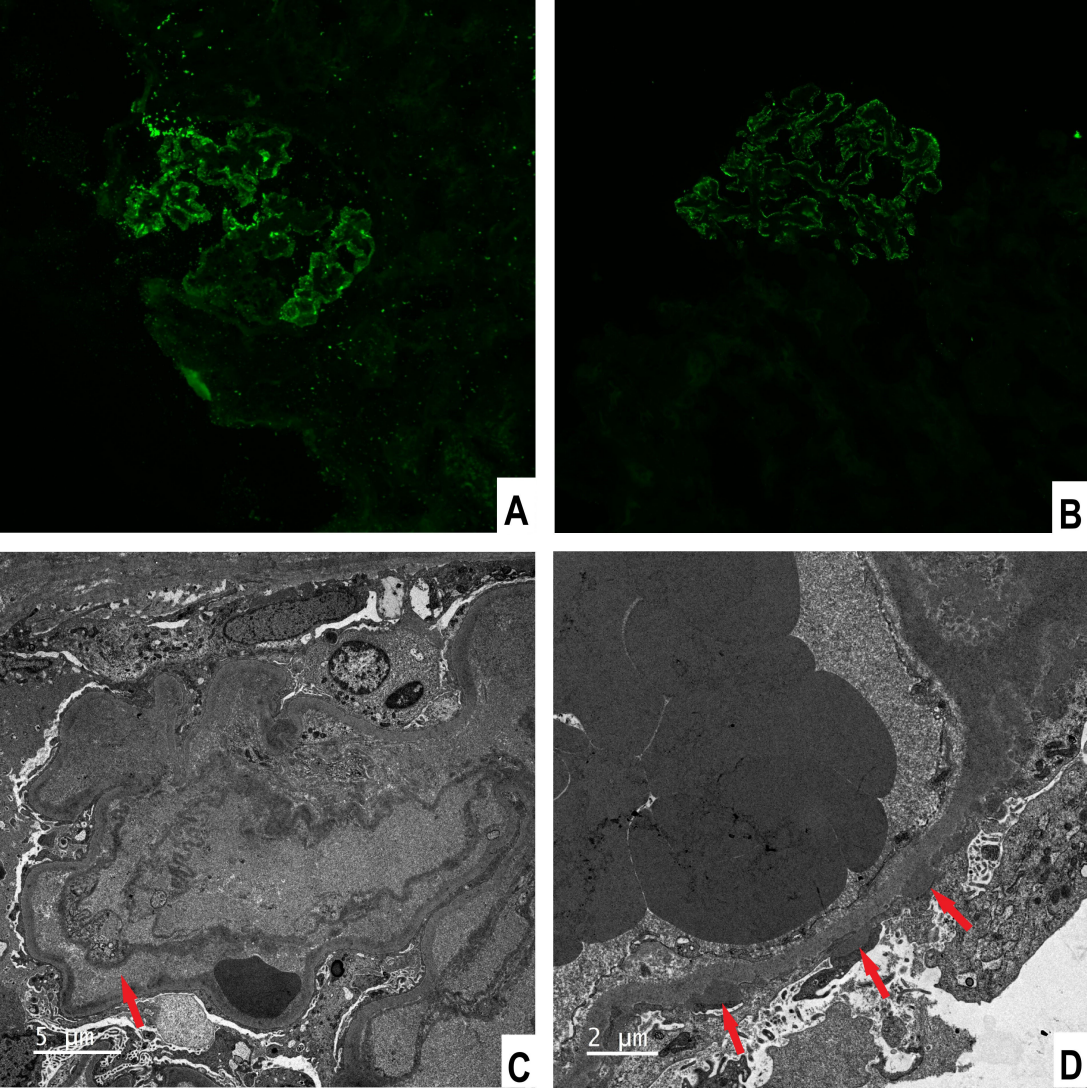
Immunofluorescence stainings and electron microscopic findings. **(A)** Fine granular staining for IgG along the glomerular tuft, × 200. **(B)** C4d staining along the glomerular tuft, × 200. **(C)** Endothelial swelling with subendothelial widening, × 4000. **(D)** Subepithelial electron-dense deposits, × 4000.

He underwent percutaneous kidney biopsy. Light microscopy showed renal thrombotic microangiopathies (TMA) and segmental spike formation (**Figure 1**). TMA was primarily chronic, as evidenced by double contour of the glomerular basement membrane and fibrous intimal hyperplasia of interlobular arteries. Endothelial swelling and intimal edema resulting in occlusion of the pre-capillary arterioles, and myxoid intimal swelling in the interlobular arteries were seldomly observed, suggesting coexistent active TMA (**Supplementary Figure S1**). Diffuse interstitial fibrosis and tubular atrophy were observed. Immunofluorescence study demonstrated focal granular IgG, diffuse C1q, and focal C4d deposits along the glomerular tuft (**Figure 2**). Staining of IgM, IgA, C3, C4, PLA2R and EBV-encoded small RNA were negative. Electron microscopic examination revealed swelling of endothelial cells, widening of subendothelial spaces, and deposits in subepithelial and mesangial areas (**Figure 2**).

His renal biopsy results revealed coexistent membranous nephropathy (MN) and renal TMA. MN secondary to chronic GVHD was considered because we found no evidence of other causes of MN, including medication, infection, connective tissue disease, and malignancy. HSCT-associated renal TMA was considered after ruling out other causes, including thrombocytopenic purpura, Shiga toxin-associated hemolytic uremic syndrome, infection, connective tissue disease, malignancy, and malignant hypertension. We could not exclude the contribution of cyclosporin and total body irradiation to the patient’s TMA, but the onset of TMA symptoms (hypertension, proteinuria, and deteriorating kidney function) after discontinuation of cyclosporin and total body irradiation suggested a possibility of GVHD-related TMA.

Liver biopsy was planned but not performed due to the high risk of bleeding evaluated by the operator. Given the concurrence of portal hypertension and the renal disease after HSCT, chronic hepatic GVHD was considered as a possible cause of portal hypertension. Hepatic venous occlusive disease was suspected initially, but the lack of hyperbilirubinemia, hepatomegaly, and typical features on contrast CT scan did not support the diagnosis. We could not exclude the role of previous EBV infection and drugs in his liver injury, but they could not explain the new-onset portal hypertension and ascites after remission of EBV infection and discontinuation of these drugs.

He was initially treated with oral prednisolone 55 mg daily which was discontinued two weeks later because examinations revealed femur head necrosis. During the two-week treatment with prednisolone, unremarkable changes were observed except a significant increase of hemoglobin (from 90 to 119 g/L), which declined to baseline after discontinuation of steroids, suggesting autoimmune-mediated anemia. Ruxolitinib 5 mg twice daily was applied from October 2022. From the 4^th^ month of treatment with ruxolitinib, the patient had partial remission of nephrotic syndrome, with improvement in kidney function, normal liver function, and remission of portal hypertension and ascites (**Supplementary Table S1**). Ruxolitinib was reduced to 5mg/day on July 2023 (9 months after treatment), but recurrence of nephrotic proteinuria after dosage reduction prompted us to restore the dosage to 5 mg twice daily (from August 2023), which led to partial remission again within 3 months. From April 2024, ruxolitinib was reduced to 5mg/d, and no recurrence was observed till the time of writing. Despite treatment with erythropoietin and iron supplement, the patient had persistent mild normocytic anemia, which was considered to be a side effect of ruxolitinib.

## Discussion

We reported a case of nephrotic syndrome along with non-cirrhotic portal hypertensive ascites after HSCT. Based on a diagnosis of exclusion, chronic GVHD was attributed as the cause of his manifestations, which were successfully treated with ruxolitinib.

Nephrotic syndrome after HSCT is uncommon with an incidence ranged from 0.4% to 6.0% (3). It usually occurs after or during tapering of immunosuppression, and has been suggested to be renal manifestation of chronic GVHD (3, 4). The most common histologic findings of post-HSCT nephrotic syndrome is MN. The presence of subepithelial deposits in post-HSCT MN suggests a role for antibody-mediated injury (3, 4), which is further supported by the resolution of MN with anti-B-cell therapy (rituximab) (3). Protocadherin FAT1 is reported to be the dominant autoantigen (5), whereas anti-PLA2R is usually negative (6, 7). Consistent with previous reports, our patient developed nephrotic syndrome after discontinuation of immunosuppression, and histological studies revealed MN with subepithelial deposits, suggesting a antibody-mediated process. The absence of serum anti-PLA2R and glomerular staining of PLA2R suggested that other autoangigen may be involved in the pathogenesis of MN, such as FAT1. However, we did not perform FAT1 staining due to unavailability of the test. Various factors may cause post-HSCT renal TMA, including irradiation, calcineurin inhibitor toxicity, infection, GVHD, etc (3). Post-HSCT renal TMA have been associated with acute or chronic GVHD previously, and most cases did not not fulfill the clinical criteria of systemic TMA (8–11). Our patient presented with renal TMA, without systemic manifestations of TMA. His renal TMA was attributed to chronic GVHD after excluding other causes. Although both post-HSCT MN and renal TMA have been associated with chronic GVHD, coexistent MN and renal TMA after HSCT was rarely reported, and we are aware of only one such case published in literature (11). C4d is a split product of C4 activation in classical and lectin pathways, serving as a marker of complement activation. Glomerular C4d deposition has been observed in various glomerular diseases in native kidneys, including MN, TMA, lupus nephritis, etc (12, 13). C4d deposition in diffuse glomerular capillaries and patchy peritubular capillaries were noted in biopsy specimens obtained from patients with post-HSCT renal TMA, suggesting a antibody-mediated process and complement activation (9–11). The kidney biopsy of our patient revealed C4d and C1q deposition along the glomerular tuft, indicating complement activation through the classical pathway.

Hepatic GVHD after 100 days can present in two ways: acute hepatitis with steeply rising alanine transaminase or a slowly progressive cholestatic disorder with elevated ALP and GGT levels (1). Our patient’s liver dysfunction manifested more like the latter presentation. Non-cirrhotic portal hypertensive ascites after HSCT was rarely reported in the literature. Most of the cases were reported in patients with venous occlusive disease (14), and a few were associated with chronic hepatic GVHD (15, 16). Cases of idiopathic portal hypertensive ascites after HSCT also exist (17). For our patient, the diagnosis of hepatic GVHD-related portal hypertension was a diagnosis of exclusion, but the lack of liver histological study precluded us to make a definite diagnosis. The histological features of chronic hepatic GVHD is progressive bile duct loss and eventually fibrosis (18). But duct loss was not observed in previously reported cases of hepatic GVHD-related portal hypertension or post-HSCT idiopathic portal hypertension (16, 17). We do not know whether bile duct injury contributed to portal hypertension in our patient. Considering the prominent endothelial injury on the patient’s renal biopsy, we hypothesized that endothelial injury of the portal vein or hepatic sinusoid secondary to GVHD might contribute to his portal hypertension. This hypothesis is supported by the suggestion that vascular endothelium is a target organ of GVHD (2, 19), and TMA is an endothelial form of GVHD (20). In addition, a recent study revealed an association between TMA and post-HSCT ascites (21), suggesting a role for endothelial injury in post-HSCT ascites.

Besides kidney and liver manifestations, the patient presented with persistent normocytic anemia after transplantation. Laboratory studies revealed positive Coombs’ test along with multiple auto-antibodies. Multiple auto-antibodies are common in patients with chronic GVHD, suggesting a pathogenic role for B cells in chronic GVHD (22). For our patient, the positive Coombs’ and the improvement of anemia after steroids therapy suggested autoimmune hemolytic anemia, which is also an atypical manifestation of chronic GVHD (2).

Immunosuppression is the primary treatment for post-HSCT MN associated with chronic GVHD. The most common treatment protocols include the combination of corticosteroids with immunosuppressive agents, and rituximab (3, 23, 24). Data on the treatment of post-HSCT TMA are limited (3), and there is no FDA approved treatment currently for post-HSCT TMA. Calcineurin inhibitors are often discontinued in the setting of post-HSCT TMA due to the association of calcineurin inhibitors with TMA. However, recent studies suggested that stopping calcineurin inhibitors might not be helpful (8), and increasing doses of calcineurin inhibitors led to resolution of TMA in a patient with TMA attributed to GVHD (25). Mii et al. reported improvement of renal function in patients with GVHD-associated TMA after increasing doses of corticosteroids (10). Benefits of eculizumab and rituximab in post-HSCT TMA were also reported, but with limited evidence (3). Information on the treatment of portal hypertensive ascites after HSCT are scarce. Tazoe et al. reported a case of portal hypertensive ascites caused by chronic GVHD, which was successfully treated with ibrutinib (16). Corticosteroid therapy was contradicted in our patient due to femur head necrosis, and calcineurin inhibitors were not chosen due to concerns of renal toxicity. We did not choose rituximab considering the risk of infection after B-cell clearance.

The pathophysiology of chronic GVHD is complex, which may involve inflammation, dysregulated T-cell and B-cell immunity, and fibrosis (26). T cell activity is the driving force behind both acute and chronic GVHD, and cytokines play a key role in promoting T cell activation, which is mediated mainly through JAK1/JAK2 (27). Ruxolitinib is a selective JAK1/JAK2 inhibitor, that has been recommended as a primary treatment in steroid-refractory chronic GVHD (28). We are aware of no previous reports on the use of ruxolitinib for post-HSCT nephrotic syndrome or portal hypertension, possibly due to the rarity of the two atypical manifestations. Based on elevated cytokines in our patient, the role of cytokines in chronic GVHD, and the efficacy of ruxolitinib in chronic GVHD through inhibition of cytokine signaling, we attempted to treat him with ruxolitinib. The treatment response suggested the efficacy of ruxolitinib for the two atypical manifestations of chronic GVHD, and a role of inflammatory cytokines in the pathogenesis of his manifestations.

There are several limitations in our case report. First, MN and kidney TMA were both attributed to chronic GVHD, which was a diagnosis of exclusion due to lack of validated diagnostic criteria for kidney GVHD. Second, the diagnosis of hepatic GVHD-related portal hypertension was a diagnosis of exclusion, but the lack of liver histological study precluded us to make a definite diagnosis and explore the underlying mechanisms. In addition, systemic cytokine activation (IL-6 and TNF-alpha) in the patient was an important reason for choosing ruxolitinib. Although ruxolitinib showed efficacy in our patient, it is worth noting that rituximab and complement inhibitors had more evidence in post-HSCT MN and TMA. More studies on how to manage post-HSCT MN and kidney TMA are required in the future.

In summary, this case highlights awareness of nephrotic syndrome and portal hypertension as atypical manifestations of chronic GVHD, and the efficacy of ruxolitinib for the two manifestations. The accumulation of further cases is required to elucidate the pathogenesis and establish the optimal treatment for nephrotic syndrome and portal hypertensive ascites after HSCT.

## Data Availability

The original contributions presented in the study are included in the article/**Supplementary Material**. Further inquiries can be directed to the corresponding authors.

## Supplementary material

Supplementary Figure 1 Light microscopic findings of arterioles and arteries. **(A)** Endothelial swelling and intimal edema resulting in occlusion of the pre-capillary arterioles, Periodic acid-schiff stain, × 200. **(B)** Myxoid intimal swelling and fibrous intimal hyperplasia in the interlobular arteries, PASM stain, × 200.

Supplementary Table 1 Result of laboratory examinations. dsDNA double strand DNA; ANCA antineutrophil cytoplasmic antibody.
