# Supplementary figures and images for: Case report: Nephrotic syndrome and portal hypertensive ascites after allogeneic hematopoietic stem cell transplantation: a rare manifestation of chronic graft-versus-host disease
Source: Front Immunol. 2024 Oct 16;15:1464616. doi: 10.3389/fimmu.2024.1464616 (PMC11521799; doi:10.3389/fimmu.2024.1464616)

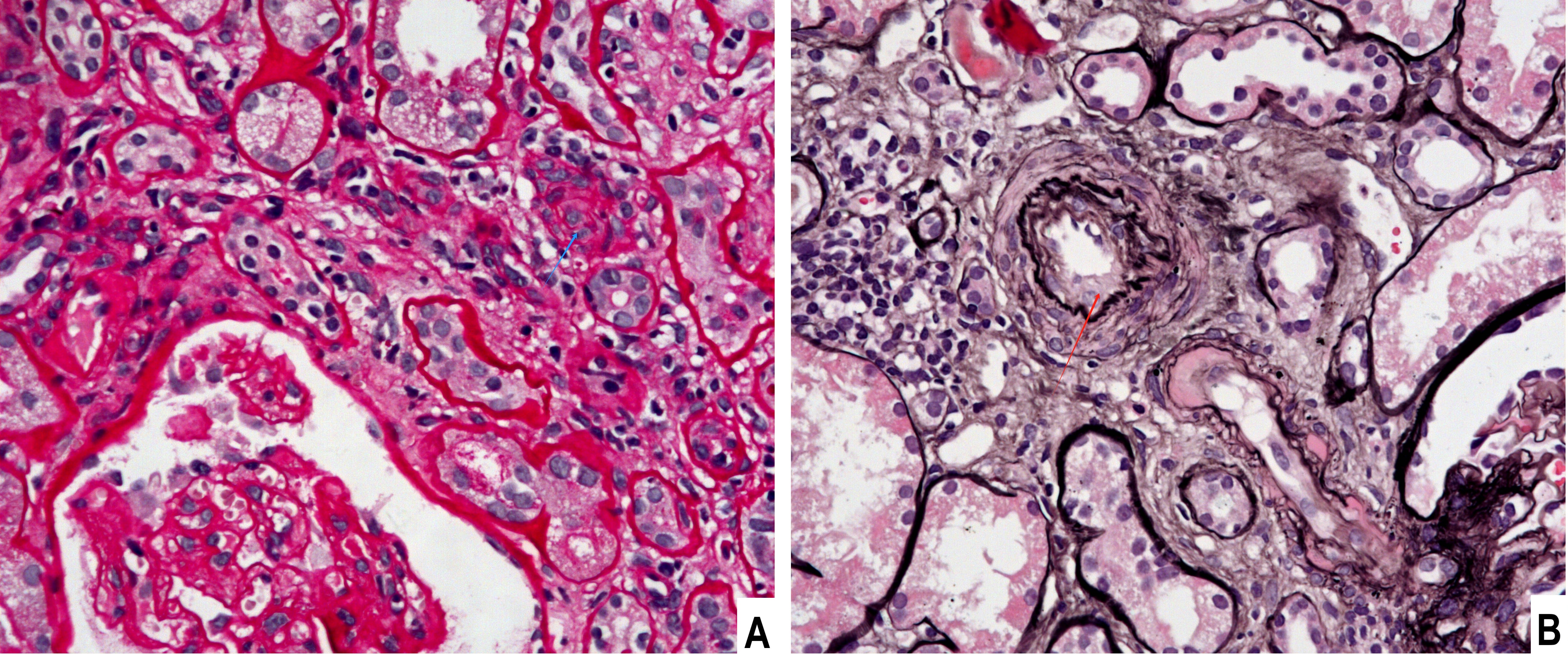

Supplement: Supplementary Figure 1 — Light microscopic findings of arterioles and arteries. (A) Endothelial swelling and intimal edema resulting in occlusion of the pre-capillary arterioles, Periodic acid-schiff stain, × 200. (B) Myxoid intimal swelling and fibrous intimal hyperplasia in the interlobular arteries, PASM stain, × 200. [file Image1.jpeg]
