# Supplementary material for: Case report: Nephrotic syndrome and portal hypertensive ascites after allogeneic hematopoietic stem cell transplantation: a rare manifestation of chronic graft-versus-host disease
Source: Front Immunol. 2024 Oct 16;15:1464616. doi: 10.3389/fimmu.2024.1464616 (PMC11521799; doi:10.3389/fimmu.2024.1464616)
Supplement: Supplementary Table 1 — Result of laboratory examinations. dsDNA double strand DNA; ANCA antineutrophil cytoplasmic antibody. [file Table1.pdf]

Table S1. Result of laboratory examinations

	On admission (August 2022)	Last Follow-up (August 2024)
**Complete blood count**		
White blood cell	4.43 × 10^9/L	5.02 × 10^9/L
Hemoglobin	92 g/L	102 g/L
Platelet	176 × 10^9/L	219 × 10^9/L
**24-hour urinary protein**	10.1 g	1.09 g
**Biochemistry**		
Albumin	29 g/L (35-52)	46 g/L
Alanine aminotransferase (ALT)	28 U/L (9-50)	27 U/L
Aspartate aminotransferase (AST)	55 U/L (15-40)	32 U/L
Total Bilirubin/ Direct Bilirubin	Normal range	Normal range
Alkaline phosphatase (ALP)	344 U/L (45-125)	114 U/L
Gamma-glutamyl transferase (GGT)	173 U/L (10-60)	32 U/L
Serum creatinine	1.80 mg/dL (0.67-1.18)	1.52 mg/dL
C-reactive protein	10.02 mg/L (< 3.00)	0.77 mg/L
**Auto-antibodies and cytokines**		
Antinuclear antibody	1: 160 (+) cytoplasmic	1: 80 (+) cytoplasmic
Anti-ds DNA (Immunofluorescence)	(-)	(-)
Anti-ds DNA (CLIA)	43 IU/ml (< 24.0)	(-)
Complement C3 and C4	Normal range	Normal range
Coombs' test	(+)	(-)
Cytoplasmic ANCA	(+) 1:20	(-)
Myeloperoxidase- and Proteinase 3-ANCA	(-)	(-)
Anti-phospholipase A2 receptor antibody	(-)	(-)
Tumor necrosis factor (TNF)-alpha	18.5 pg/ml (< 8.1)	13.4 pg/ml (<15.2)
Interleukin-6	13.4 pg/ml (< 5.9)	2.4 pg/ml (<5.9)
Interleukin-8 and 10	Normal range	Normal range

dsDNA double strand DNA; ANCA antineutrophil cytoplasmic antibody.
